# Prognostic impact of chromosome aberrations in ovarian cancer.

**DOI:** 10.1038/bjc.1992.56

**Published:** 1992-02

**Authors:** T. Pejovic, A. Himmelmann, S. Heim, N. Mandahl, U. M. Flodérus, S. Furgyik, B. Elmfors, G. Helm, H. Willén, F. Mitelman

**Affiliations:** Department of Clinical Genetics, University Hospital, Lund, Sweden.

## Abstract

Clinico-cytogenetic correlations were assessed in 88 patients with malignant ovarian tumours. Cytogenetic analysis of the primary tumours yielded normal karyotype (N) in 33 patients and abnormal karyotypes (A) in 55 patients. Within the A group, seven tumours had simple abnormalities (AS), i.e., numerical changes only or a single structural aberration, and 48 had karyotypes with complex aberrations (AC). A correlation analysis between groups N and A revealed that cytogenetic abnormalities were more often found among seropapillary tumours, and that cases with abnormal karyotypes on average were of higher stage and more often had residual tumour mass after initial surgery (P less than 0.05 for all variables). When the three groups N, AS, and AC were compared, they were found to be significantly different with regard not only to the three parameters mentioned above, but now tumour grade also appeared to correlate with karyotypic pattern (P = 0.001), with poorly differentiated tumours having the most complex karyotypes. In a correlation analysis between karyotypic pattern and survival, group A patients had shorter survival than group N (P = 0.049). In the corresponding analysis between groups N, AS, and AC, the differences were also significant (P = 0.039), with shorter survival in group AC than in groups N and AS. Stage, grade, residual tumour after primary surgery, and performance status also correlated with survival time. A multivariate analysis identified abnormal karyotype as being independently associated with short survival in advanced clinical stages (P = 0.030) of ovarian carcinoma. We conclude that cytogenetic analysis of tumour cells may be of clinical value in the assessment of prognosis in patients with malignant ovarian tumours.


Br. J. Cancer (1992), 65, 282-286 © Macmillan Press Ltd., 1992

### Prognostic impact of chromosome aberrations in ovarian cancer

T. Pejovic[1], A. Himmelmann[2], S. Heim[1,7], N. Mandahl[1], U.-M. Flodérus[3], S. Furgyik[5], B. Elmfors[6], G. Helm[6], H. Willén[4] & F. Mitelman[1]

*Departments of [1]Clinical Genetics, [2]Gynecological Oncology, [3]Gynecology, and [4]Pathology, University Hospital, Lund, Sweden; [5]Department of Gynecology, General Hospital, Malmö, Sweden; [6]Department of Gynecology, Central Hospital, Kristianstad, Sweden; and [7]Department of Medical Genetics, Odense University, Odense, Denmark.*

**Summary** Clinico-cytogenetic correlations were assessed in 88 patients with malignant ovarian tumours. Cytogenetic analysis of the primary tumours yielded normal karyotype (N) in 33 patients and abnormal karyotypes (A) in 55 patients. Within the A group, seven tumours had simple abnormalities (AS), i.e., numerical changes only or a single structural aberration, and 48 had karyotypes with complex aberrations (AC). A correlation analysis between groups N and A revealed that cytogenetic abnormalities were more often found among seropapillary tumours, and that cases with abnormal karyotypes on average were of higher stage and more often had residual tumour mass after initial surgery ($P < 0.05$ for all variables). When the three groups N, AS, and AC were compared, they were found to be significantly different with regard not only to the three parameters mentioned above, but now tumour grade also appeared to correlate with karyotypic pattern ($P = 0.001$), with poorly differentiated tumours having the most complex karyotypes. In a correlation analysis between karyotypic pattern and survival, group A patients had shorter survival than group N ($P = 0.049$). In the corresponding analysis between groups N, AS, and AC, the differences were also significant ($P = 0.039$), with shorter survival in group AC than in groups N and AS. Stage, grade, residual tumour after primary surgery, and performance status also correlated with survival time. A multivariate analysis identified abnormal karyotype as being independently associated with short survival in advanced clinical stages ($P = 0.030$) of ovarian carcinoma. We conclude that cytogenetic analysis of tumour cells may be of clinical value in the assessment of prognosis in patients with malignant ovarian tumours.

Despite recent improvements in the treatment of ovarian carcinoma patients, tumour- and host-related factors still seem to determine the outcome to a much larger degree than does the therapy (Swenerton *et al.*, 1985; van Houwelingen *et al.*, 1989). Clinical stage, the size of the postoperative residual tumour mass, the tumour's histologic type and grade, and the patient's age and performance status have in numerous retrospective analyses been identified as prognostic factors, but their interrelationship is complex and their combined predictive power is often weak (Marsoni *et al.*, 1990).

In addition to these partly subjective clinical and pathologic parameters, several other potential prognostic factors have also been studied in ovarian carcinoma patients. These include steroid receptor status (Slotman *et al.*, 1990), *in vitro* chemosensitivity (Volm *et al.*, 1985), level of CA125 antigen (Rustin *et al.*, 1989), DNA content as measured by flow cytometry (Friedlander *et al.*, 1988; Iversen, 1988; Brescia *et al.*, 1990), clonogenic growth *in vitro* (Dittrich *et al.*, 1991), oncogene expression (Berchuck *et al.*, 1990), and growth factor receptor expression (Bauknecht *et al.*, 1989; Berchuck *et al.*, 1991). However, the significance of these parameters as independent prognostic factors remains unclear.

The karyotype has been demonstrated to be of prognostic value in patients with acute myeloid leukaemia (Arthur *et al.*, 1989), acute lymphoblastic leukaemia (Bloomfield *et al.*, 1989), myelodysplastic disorders (Pierre *et al.*, 1989), and non-Hodgkins lymphoma (Fifth International Workshop on Chromosomes in Leukemia-Lymphoma, 1987). Because the cytogenetic data base is so much more limited for non-haematologic neoplasms, only few attempts have hitherto been made to correlate karyotypic findings with clinical parameters in patients with solid tumours (e.g., Rydholm *et al.*, 1990; Trent *et al.*, 1990; Lundgren *et al.*, 1991). We examined whether such correlations could be found in ovarian carcinoma patients.

Correspondence: T. Pejovic, Department of Clinical Genetics, University Hospital, S-221 85 Lund, Sweden.
Received 17 June 1991; and in revised form 7 October 1991.

### Materials and methods

### *Patients*

During 28 months in 1988-1990 successful cytogenetic analysis after short-term culture was performed on tumour samples from 88 previously untreated patients with primary malignant tumours of the ovary (Table I). All samples were obtained at the time of initial surgery. The tumours were classified histologically using standard WHO criteria (Serov *et al.*, 1973). Although mixed mesodermal tumours have been classified by WHO as common epithelial tumours, they contain both carcinomatous and sarcomatous components (Clarke, 1990) and the four tumours of this type were grouped separately (Table I). The carcinomas were histologically graded as well differentiated, moderately differentiated, and poorly differentiated (Czernobilsky, 1987). Five borderline carcinomas were also included in the study. Staging (FIGO, 1988) was based on the findings at surgical exploration.

The mean age of the patients at the time of surgery and cytogenetic analysis was 62.5 years (range 24-87 years). Sixty-five patients were in good general condition (Karnofsky index 90-100), whereas 23 patients had poorer performance status (Karnofsky index $\leqslant 80$). The patients were monitored regularly after primary surgery and clinical data were collected until death or March 1, 1991. Median follow-up time from surgery was 12.5 months for the entire group (range 2.5-26.8 months). The median follow-up time for the groups with normal, abnormal, simple, and complex karyotypes (see below) was 13.5, 10.0, 8.8 and 11.0 months, respectively.

Following informed consent, 73 of the 88 patients received combination chemotherapy after surgery: cisplatin + cyclophosphamid (41 patients), other cisplatin-based combinations (15 patients), and doxorubicin + melphalan (17 patients). After the four prescribed courses of chemotherapy, 34 patients underwent second-look laparotomy to determine the response to therapy.

### *Cytogenetic methods*

The culturing, harvesting, and banding techniques for chromosome analysis have been described in detail (Pejovic

**Table I** Karyotypic pattern in relation to clinico-pathologic factors of prognostic importance in 88 patients with ovarian cancer

| Prognostic factors | *Karyotype* Normal (N) n = 33 | *Abnormal (A)* Simple (AS) n = 7 | *Abnormal (A)* Complex (AC) n = 48 | *P-value* N vs A | *P-value* N vs As and AC |
|---|---|---|---|---|---|
| Grade[a] | | | | | |
| Well | 6 | 5 | 0 | 0.226 | 0.001 |
| Moderate | 10 | 0 | 16 | | |
| Poor | 11 | 1 | 28 | | |
| Borderline tumours | 4 | 1 | 0 | | |
| Histologic subtype | | | | | |
| Seropapillary | 13 | 4 | 35 | 0.017 | 0.033 |
| Mucinous | 7 | 2 | 0 | | |
| Endometrioid | 6 | 1 | 7 | | |
| Clear cell | 5 | 0 | 2 | | |
| Undifferentiated | 1 | 0 | 1 | | |
| Mixed mesodermal | 1 | 0 | 3 | | |
| Stage (FIGO) | | | | | |
| I | 15 | 3 | 4 | 0.003 | 0.004 |
| II | 5 | 1 | 5 | | |
| III | 10 | 3 | 28 | | |
| IV | 3 | 0 | 11 | | |
| Residual tumour after initial surgery | | | | | |
| Present | 12 | 2 | 41 | 0.001 | 0.001 |
| Not present | 21 | 5 | 7 | | |
| Age | | | | | |
| $<50$ | 3 | 1 | 6 | 0.122 | 0.264 |
| 50–69 | 16 | 4 | 32 | | |
| $\geqslant 70$ | 14 | 2 | 10 | | |
| Performance status (Karnofsky index) | | | | | |
| 90–100 | 27 | 6 | 32 | 0.188 | 0.237 |
| $\leqslant 80$ | 6 | 1 | 16 | | |

[a]Borderline tumours, mixed mesodermal tumours, and undifferentiated carcinomas are not included.

*et al.*, 1989). Briefly, the tumour samples were minced with scissors, disaggregated enzymatically, and transferred onto glass chamber slides in RPMI 1640 medium with mitogenic additives. After 2–6 days the cultures were exposed to Colcemid and harvested by hypotonic treatment and repeated fixations. G-banding was obtained with Wright's stain. All clonal aberrations (ISCN, 1985) were identified in at least two different *in situ* preparations.

For the various correlation analyses, the 88 cases were broken down into two main categories – cases with normal (N) *vs* cases with abnormal (A) karyotype. The latter group was subdivided depending on whether the karyotype contained complex (AC) or simple (AS) abnormalities; simple abnormalities were defined as numerical changes only or a single structural rearrangement (Pejovic *et al.*, 1991*a*). Another subdivision was based on the modal chromosome number and stratified the abnormal clones into four groups: hypodiploid (35–45 chromosomes), hyperdiploid (47–57 chromosomes), near-triploid (58–80 chromosomes), and near-tetraploid (81–103 chromosomes). The two cases with a pseudodiploid tumour karyotype were excluded from the modal number-based analysis. The third subdivision was according to the presence or absence of structural aberrations involving chromosome arms 3p, 6q, 11p, 19p, and 19q, all of which have been described as nonrandomly associated with ovarian carcinoma (Pejovic *et al.*, 1991*a*).

The detailed abnormal karyotypes of all but two mixed mesodermal and one borderline tumour have been published (Pejovic *et al.*, 1989; 1990*a,b*; 1991*a,b*). Thirty-three of the 88 tumours had a normal karyotype (N) and 55 had an abnormal karyotype (A). Of the 55 aberrant tumours, seven had simple karyotypic changes (AS), the remaining 48 had complex karyotypes (AC) with multiple structural and numerical abnormalities. Within the A group, 10 tumours had a hypodiploid chromosome number, nine were hyperdiploid, 28 were near-triploid, and six tumours had a near-tetraploid modal chromosome number. The most frequent structural aberrations – involving at least one-fourth of tumours with complex karyotype changes – affected chromosome arms 3p (17 tumours), 6q (16 tumours), 11p (20 tumours), 19p (25 tumours), and 19q (12 tumours).

### *Statistical methods*

Covariation between the karyotype and known clinico-pathologic prognostic factors (Table I) was estimated using the Chi-square test. The potential prognostic importance of karyotypic changes was assessed by comparing survival times from surgery to death according to Kaplan and Meier (1958). The generalised Wilcoxon's statistics was used in univariate analyses of possible prognostic factors (Tables II and III). Analysis of prognostic factors with simultaneous evaluation of their relative importance was performed by the Cox (1972) regression model (Table IV). The potential covariation of the different variables with the achievement of complete response as the result of the primary treatment was estimated by the Chi-square test.

### Results

The number of patients with normal (N) and abnormal (A) karyotypes, in relation to tumour stage, histologic subtype and grade, presence of residual tumour after initial surgery, age, and performance status, is given in Table I. An abnormal karyotype was more often found in seropapillary tumours ($P = 0.017$), in more advanced (FIGO stage III–IV) disease ($P = 0.003$), and A cases also more often had residual tumour after primary surgery ($P = 0.001$). Splitting the group A into AS and AC did not markedly change the results, except that also tumour grade now emerged as a factor showing significant ($P = 0.001$) covariation with the karyo-

**Table II** Survival in relation to potential clinico-pathologic prognostic factors in 88 patients with ovarian cancer

| *Prognostic factor* | *No. of patients* | *No. of deaths* | *P-value* |
|---|---|---|---|
| Grade | | | |
| Well | 11 | 0 | 0.030 |
| Moderate | 26 | 2 | |
| Poor | 40 | 10 | |
| Histologic subtype | | | |
| Seropapillary | 52 | 11 | 0.170 |
| Mucinous | 9 | 0 | |
| Endometrioid | 14 | 0 | |
| Clear cell | 7 | 1 | |
| Undifferentiated | 2 | 0 | |
| Mixed mesodermal | 4 | 2 | |
| Stage | | | |
| I | 22 | 1 | 0.001 |
| II | 11 | 0 | |
| III | 41 | 7 | |
| IV | 14 | 6 | |
| Residual tumour after initial surgery | | | |
| Present | 55 | 13 | 0.011 |
| Not present | 33 | 1 | |
| Age | | | |
| <50 | 26 | 1 | 0.186 |
| 50–69 | 52 | 6 | |
| ⩾70 | 26 | 7 | |
| Performance status (Karnofsky index) | | | |
| 90–100 | 65 | 4 | 0.001 |
| ⩽80 | 23 | 10 | |

**Table III** Survival in relation to karyotypic pattern, modal chromosome number, and type of structural chromosome aberrations in patients with ovarian cancer

| *Karyotypic feature* | *No. of patients* | *No. of deaths* | *P-value* |
|---|---|---|---|
| Karyotype | | | |
| Normal | 33 | 2 | 0.0496 |
| Abnormal | 55 | 12 | |
| Normal | 33 | 2 | 0.039 |
| Abnormal-simple | 7 | 0 | |
| Abnormal-complex | 48 | 12 | |
| Modal chromosome number | | | |
| Hypodiploid | 11 | 2 | 0.199 |
| Hyperdiploid | 12 | 1 | |
| Near-triploid | 24 | 7 | |
| Near-tetraploid | 6 | 2 | |
| Structural aberrations | | | |
| 3p changes | 17 | 4 | 0.620 |
| no 3p changes | 31 | 8 | |
| 6q changes | 16 | 2 | 0.194 |
| no 6q changes | 32 | 10 | |
| 11p changes | 20 | 6 | 0.486 |
| no 11p changes | 28 | 6 | |
| 19p changes | 26 | 7 | 0.649 |
| no 19p changes | 22 | 5 | |
| 19q changes | 13 | 3 | 0.837 |
| no 19q changes | 35 | 9 | |

typic pattern (the tumours of AC cases were often poorly differentiated, while the N and AS tumours showed higher degrees of differentiation).

The correlation between survival and various clinico-pathologic potential prognostic parameters is shown in Table II. Group differences in survival time were observed when patients were classified according to tumour grade, stage, presence of residual tumour mass, and performance status ($P < 0.05$ for all variables, Table II, Figure 1). Histologic subtype and patient age did not correlate with survival.

The correlation between survival and karyotype is given in Table III. The A patients had shorter survival than N patients ($P = 0.0496$, Table III, Figure 2a). After dividing the

**Table IV** Cox regression analysis showing the most important prognostic factors in 52 ovarian carcinoma cases of FIGO stages III–IV

| *Prognostic factor*[a] | *P-value* | *Relative risk*[b] |
|---|---|---|
| Age⩾60 (<60 = 1) | 0.003 | 8.485 |
| Grade – poor (well + moderate = 1) | 0.004 | 19.120 |
| Karyotype – abnormal (normal = 1) | 0.030 | 6.865 |
| Performance status (Karnofsky index) ⩽80 (90 – 100 = 1) | 0.051 | 3.405 |

[a]The table represents step 1 of the regression analysis; tumur stage has been removed from the analysis because it did not appear as an independent prognostic parameter ($P = 0.917$) in step 0. [b]Relative risk of dying represents the hazard rate associated with a given factor relative to the most favourable condition ( = 1) for the same factor.

**Figure 1** Survival in 88 ovarian cancer patients in relation to histologic grade, FIGO stage, residual tumour after initial surgery, and performance status.

series into the three cytogenetic groups N, AS and AC in the correlation analysis, the difference remained statistically significant ($P = 0.039$), and the AC group exibited the shortest survival (Figure 2b).

Tumour grade, clinical stage, age, and performance status were factors used as covariates in the Cox proportional hazard model to evaluate if karyotypic changes could predict outcome independently (Table IV). Because there were so few deaths among stages I–II, only information about the 52 patients with advanced disease (stages III–IV) was used in the multivariate analysis. Only three patients in this group were without residual tumour after primary surgery, and therefore this factor was not considered in the analysis. Finally, the three mixed mesodermal tumours of stages III–IV were excluded from the analysis because they are regarded to constitute an entity that is separate from the true epithelial ovarian tumours. Most carcinomas (43 of the 52) were of the seropapillary type. Tumour grade, age, and karyotype provided independent prognostic information on survival in this series. The prognosis was worse for patients with karyotypic abnormalities (all but one belonged to the AC group) compared with those with normal karyotypes ($P = 0.030$). Similarly, poorly differentiated tumours and older age were associated with short survival ($P = 0.004$ and $P = 0.003$, respectively). The correlation between performance status and survival was borderline ($P = 0.051$).

**Figure 2** Survival in 88 ovarian cancer patients with **a**, normal (N) and abnormal (A) tumour karyotypes and **b**, normal karyotype (N), abnormal karyotype with simple aberrations (AS), and abnormal karyotype with complex aberrations (AC).

Clinical stage III *vs* IV did not show any influence on survival.

When the presence of particular structural aberrations was correlated with tumour stage, presence of residual tumour after initial surgery, histologic subtype and grade, patient's age and performance status (data not shown), the occurrence of 3p changes (in 17 tumours) showed a correlation with poor performance status ($P = 0.027$) and the 11p changes (in 20 tumours) were more frequent in cases of advanced clinical stage ($P = 0.047$). No difference in survival time between patients with any particular structural aberration and those without was found (Table III). Other comparisons between the groups with different structural aberrations could not be performed because some tumours had simultaneously several of the chromosome anomalies. There was also no difference in survival time when groups with different modal chromosome numbers were compared (Table III).

The relationship between karyotype – as well as the other clinico-pathologic factors – and response to primary treatment could be evaluated in the 34 patients (seven with early and 27 with advanced disease stage) who underwent second-look operations. The only variable that was significantly associated with achievement of complete remission was histologic tumour grade ($P = 0.014$). All three patients with well differentiated tumours achieved complete remission, whereas eight of the 16 patients with moderately differentiated tumours and only two of the 14 patients with poorly differentiated tumours had microscopically documented complete remission. One mixed mesodermal tumour was not graded.

### Discussion

Some cytogenetic studies performed on unbanded (Atkin, 1971) as well as banded material (Whang-Peng *et al.*, 1984; Trent *et al.*, 1985) have hinted that the degree of chromosome alteration in ovarian carcinomas may influence patient survival. This report is the first to statistically examine the potential covariation between tumour karyotype and known important clinico-pathologic parameters, and the influence of all these potential prognostic factors on the response to primary treatment and the survival of the patient.

Our rationale for subdividing patients with abnormal (A) karyotypes into two subgroups, those with simple (AS) and those with complex (AC) changes, was our earlier observation that simple karyotypic changes are much more common in well differentiated ovarian carcinomas than in moderately and poorly differentiated tumours (Pejovic *et al.*, 1990*b*, 1991*a*). Both in the N *vs* A analysis and in the N *vs* AS and AC comparisons the karyotypic pattern showed correlation with tumour stage, histologic type, and age of the patient. However, only when the A group was subdivided into AS and AC were the histologic tumour grade and karyotypic pattern significantly correlated (Table I): well differentiated carcinomas tended to have normal or simple karyotypes, whereas poorly differentiated tumours tended to have complex karyotypes. Also, tumours of stages III–IV more often had complex karyotypes than tumours of early stages. Similary, patients with N or AS tumour karyotypes were more frequently tumour-free after the initial operation than AC patients. All these results are in agreement with the generally accepted view of gradual accumulation of genetic alterations during tumour progression, with more malignant tumours having more aberrant karyotypes (Nowell, 1976). Complex karyotypes were also more frequently found among seropapillary carcinomas, whereas mucinous and clear-cell carcinomas tended to have normal karyotypes.

Of the subsets defined by the presence of particular structural chromosomal abnormalities, only tumours with 11p changes showed a correlation with advanced clinical stage and tumours with 3p aberrations correlated with poor performance status. These associations could well be spurious and reflect chance significances rather than reproducible biological mechanisms. There was no association between a 19p+ chromosome, the most frequent structural aberration in our series, and any of the prognostic parameters. The presence of similar 19p+ chromosomes has in patients with malignant fibrous histiocytoma been associated with increased risk of relapse (Rydholm *et al.*, 1990).

The factors generally accepted as predicting survival length in ovarian carcinoma patients (Swenerton *et al.*, 1985) were shown to be of importance also in our series: grade, stage, residual tumour, and performance status were all significantly correlated with survival (Table II, Figure 1). No similar correlation was found for histologic type, which is in agreement with reports stressing that grading provides more prognostic information than histopathologic diagnosis in ovarian carcinoma patients (Sorbe *et al.*, 1982; Malkasian *et al.*, 1984). The degree of cytogenetic complexity was strongly correlated with outcome: not only did patients with abnormal karyotypes have shorter survival than those with normal karyotypes, but also within the A group the AC cases had shorter survival than the AS cases (Table III, Figure 2a,b). On the other hand, no difference in survival was apparent between patients with normal tumour karyotypes and those belonging to the AS subset. It is at present uncertain whether this reflects favourable prognosis for the AS group or whether the similarity would disappear with longer observation time and when more patients are examined. In the multivariate analysis (Table IV), abnormal karyotype was shown to be an independent prognostic discriminator for patients with advanced (stage III–IV) seropapillary ovarian carcinomas, though not with higher predictive power than grade and age. The findings are in accordance with previous flow cytometric studies showing that an aneuploid DNA content in the tumours has an adverse effect on the survival of the patient (Friedlander *et al.*, 1988; Iversen, 1988; Klemi *et al.*, 1988; Brescia *et al.*, 1990).

The only cytogenetic parameter that in previous studies was suggested to be of prognostic importance in ovarian carcinoma patients was the modal chromosome number: Trent *et al.* (1985) found correlation between hypodiploid, and particularly near-haploid, ovarian cancers and poor outcome. There was no near-haploid tumour present in our series, but in general we saw no difference in survival between different ploidy groups.

In spite of a short follow-up time and relatively small number of patients in our study, the tumour karyotype could nevertheless, for the first time, be demonstrated to be an independent prognostic parameter in ovarian cancer patients. But even if the finding of a normal or minimally altered karyotype indicates that an ovarian cancer patient has a relatively favourable prognosis, it is apparent that not all these cases follow an indolent clinical course. This may for some tumours be explained by our failure to detect cytogeneticially the abnormal clones. Any tumour sample consists of a mixture of parenchyma and stroma cells, and preferential outgrowth of the latter leads to the impression of a normal tumour karyotype although the parenchymal elements may have harboured abnormalities. Improvement of the tissue culturing techniques as well as the combined use of cytogenetic and flow cytometric analyses may in the future result in better identification of patients with favourable prognosis.

We thank Jonas Ranstam for help with the statistical analyses. The study was financially supported by the Swedish Cancer Society, the Lund University Medical Faculty, and the JAP Foundation for Medical Research.

### References

ARTHUR, D.C., BERGER, R., GOLOMB, H.M. & 16 others (1989). The clinical significance of karyotype in acute myelogenous leukemia. *Cancer Genet. Cytogenet.*, **40**, 203.

ATKIN, N.B. (1971). Modal DNA value and chromosome number in ovarian neoplasia. *Cancer*, **27**, 1064.

BAUKNECHT, T., JANZ, I. & KOHLER, M. (1989). Correlation of malignancy and survival with the expression of epidermal growth factor receptors (EGF-R) and EGF-like factors (EGF-F). *Med. Oncol. Tumor Pharmacother.*, **6**, 121.

BERCHUCK, A., KAMEL, A., WHITAKER, R. & 10 others (1990). Overexpression of *HER-2/neu* is associated with poor survival in advanced epithelial ovarian cancer. *Cancer Res.*, **50**, 4087.

BERCHUCK, A., RODRIGUEZ, G.C., KAMEL, A. & 4 others (1991). Epidermal growth factor receptor expression in normal ovarian epithelium and ovarian cancer. *Am. J. Obstet. Gynecol.*, **164**, 669.

BLOOMFIELD, C.D., SECKER-WALKER, L.M., GOLDMAN, A.I. & 14 others (1989). Six-year follow-up of the clinical significance of karyotype in acute lymphoblastic leukemia. *Cancer Genet Cytogenet.*, **40**, 171.

BRESCIA, R.J., BARAKAT, R.A., BELLER, U. & 4 others (1990). The prognostic significance of nuclear DNA content in malignant epithelial tumors of the ovary. *Cancer*, **65**, 141.

CLARKE, T.J. (1990). Histogenesis of ovarian malignant mixed mesodermal tumours. *J. Clin. Pathol.*, **43**, 287.

COX, D.R. (1972). Regression models and life-tables. *J. R. Stat. Soc. (B)*, **34**, 187.

CZERNOBILSKY, B. (1987). Common epithelial tumors of the ovary. In *Blaustein's Pathology of the Female Genital Tract*, Kurman, R.J. (ed.), pp. 560–606. Springer-Verlag: New York.

DITTRICH, C., DITTRICH, E., SEVELDA, P. & 4 others (1991). Clonogenic growth in vitro: an independent biologic prognostic factor in ovarian carcinoma. *J. Clin. Oncol.*, **9**, 381.

FIFTH INTERNATIONAL WORKSHOP ON CHROMOSOMES IN LEUKEMIA-LYMPHOMA (1987). Correlation of chromosome abnormalities with histologic and immunologic characteristics in non-Hodgkin's lymphoma and adult T cell leukemia-lymphoma. *Blood*, **70**, 1554.

FIGO (1988). Annual report on the results of treatment in gynecological cancer. Vol. 20. Radiumhemmet: Stockholm, Sweden.

FRIEDLANDER, M.L., HEDLEY, D.W., SWANSON, C. & RUSSELL, P. (1988). Prediction of long-term survival by flow cytometric analysis of cellular DNA content in patients with advanced ovarian cancer. *J. Clin. Oncol.*, **6**, 282.

ISCN (1985). *An International System for Human Cytogenetic Nomenclature*. Harnden, D.G. & Klinger, H.P. (eds). Published in collaboration with Cytogenet Cell Genet. Basel: Karger. (Also in Birth Defects: original article series, Vol 21, No. 1. New York: March of Dimes Birth Defects Foundation).

IVERSEN, O.-E. (1988). Prognostic value of the flow cytometric DNA index in human ovarian carcinoma. *Cancer*, **61**, 971.

KAPLAN, E.L. & MEIER, P. (1958). Nonparametric estimation from incomplete observations. *J. Am. Stat. Assoc.*, **53**, 457.

KLEMI, P., JOENSUU, H., KIILHOLMA, P. & MÄENPÄÄ, J. (1988). Clinical significance of abnormal nuclear DNA content in serous ovarian tumors. *Cancer*, **62**, 2005.

LUNDGREN, R., HEIM, S., MANDAHL, N., ANDERSON, H. & MITELMAN, F. (1991). Chromosome abnormalities are associated with unfavorable outcome in prostatic cancer patients. *J. Urol.* (in press).

MALKASIAN, G.D. Jr, MELTON, L.J., O'BRIEN, P.C. & GREENE, M.H. (1984). Prognostic significance of histologic classification and grading of epithelial malignancies of the ovary. *Am. J. Obstet. Gynecol.*, **149**, 274.

MARSONI, S., TORRI, V., VALSECCHI, M.G. & 14 others (1990). Prognostic factors in advanced epithelial ovarian cancer. *Br. J. Cancer*, **62**, 444.

NOWELL, P.C. (1976). The clonal evolution of tumor cell populations. *Science*, **194**, 23.

PEJOVIC, T., HEIM, S., MANDAHL, N. & 6 others (1989). Consistent occurrence of a 19p+ marker chromosome and loss of 11p material in ovarian seropapillary cystadenocarcinomas. *Genes. Chrom. Cancer*, **1**, 167.

PEJOVIC, T., HEIM, S., MANDAHL, N., FLODÉRUS, U.-M., WILLÉN, H. & MITELMAN, F. (1990*a*). Complex karyotypic anomalies, including an i(5p) marker chromosome, in a malignant mixed mesodermal tumor of the ovary. *Cancer Genet. Cytogenet.*, **46**, 65.

PEJOVIC, T., HEIM, S., ÖRNDAL, C. & 4 others (1990*b*). Simple numerical changes in well-differentiated malignant epithelial tumors. *Cancer Genet. Cytogenet.*, **49**, 95.

PEJOVIC, T., HEIM, S., MANDAHL, N. & 7 others (1991*a*). Chromosome aberrations in 35 primary ovarian carcinomas. *Genes Chrom. Cancer* (in press).

PEJOVIC, T., HEIM, S., MANDAHL, N. & 6 others (1991*b*). Bilateral ovarian carcinoma – cytogenetic evidence of unicentric origin. *Int. J. Cancer*, **47**, 358.

PIERRE, R.V., CATOVSKY, D., MUFTI, G.J. & 12 others (1989). Clinical-cytogenetic correlations in myelodysplasia (preleukemia). *Cancer Genet. Cytogenet.*, **40**, 149.

RUSTIN, G.J.S., GENNINGS, J.N. & NILSTROP, A.E. (1989). Use of CA125 to predict survival of patients with ovarian carcinoma. *J. Clin. Oncol.*, **7**, 1667.

RYDHOLM, A., MANDAHL, N., HEIM, S., KREICBERGS, A., WILLÉN, H. & MITELMAN, F. (1990). Malignant fibrous histiocytomas with a 19p+ marker chromosome have increased relapse rate. *Genes Chrom. Cancer*, **2**, 296.

SEROV, F., SCULLY, R.E. & SOBIN, L.E. (1973). Histological typing of ovarian tumors. In *International Classification of Tumors*. World Health Organization: Geneva.

SLOTMAN, B.J., NAUTA, J.P. & RAO, B.R. (1990). Survival of patients with ovarian cancer. Apart from stage and grade, tumor progesterone receptor content is a prognostic indicator. *Cancer*, **66**, 740.

SORBE, B., FRANKENDAL, B. & VERESS, B. (1982). Importance of histologic grading in the prognosis of epithelial ovarian carcinoma. *Obstet. Gynecol.*, **59**, 567.

SWENERTON, K.D., HISLOP, T.G., SPINELLI, J., LERICHE, J.C., YANG, N. & BOYES, D.A. (1985). Ovarian carcinoma: a multivariate analysis of prognostic factors. *Obstet. Gynecol.*, **65**, 264.

TRENT, J.M. (1985). Prevalence and clinical significance of cytogenetic abnormalities in human ovarian cancer. In *Ovarian Cancer*. Alberts, D.S. & Surwit, E.A. (eds), pp. 1–21. Martinus Nijhoff Publishers: Boston.

TRENT, J.M., MEYSKENS, F.L., SALMON, S.E. & 4 others (1990). Relation of cytogenetic abnormalities and clinical outcome in metastatic melanoma. *N. Engl. J. Med.*, **322**, 1508.

VAN HOUWELINGEN, J.C., TEN BOKKEL HUININK, W.W., VAN DER BURG, M.E.L., VAN OOSTEROM, A.T. & NEIJT, J.P. (1989). Predictability of the survival of patients with advanced ovarian cancer. *J. Clin. Oncol.*, **7**, 769.

VOLM, M., BRÜGGEMANN, A. & GÜNTHER, M. (1985). Prognostic relevance of ploidy, differentiation, and resistance-predictive tests in ovarian carcinoma. *Cancer Res.*, **45**, 5180.

WHANG-PENG, J., KNUTSEN, T., DOUGLASS, E.C. & 4 others (1984). Cytogenetic studies in ovarian cancer. *Cancer Genet. Cytogenet.*, **11**, 91.


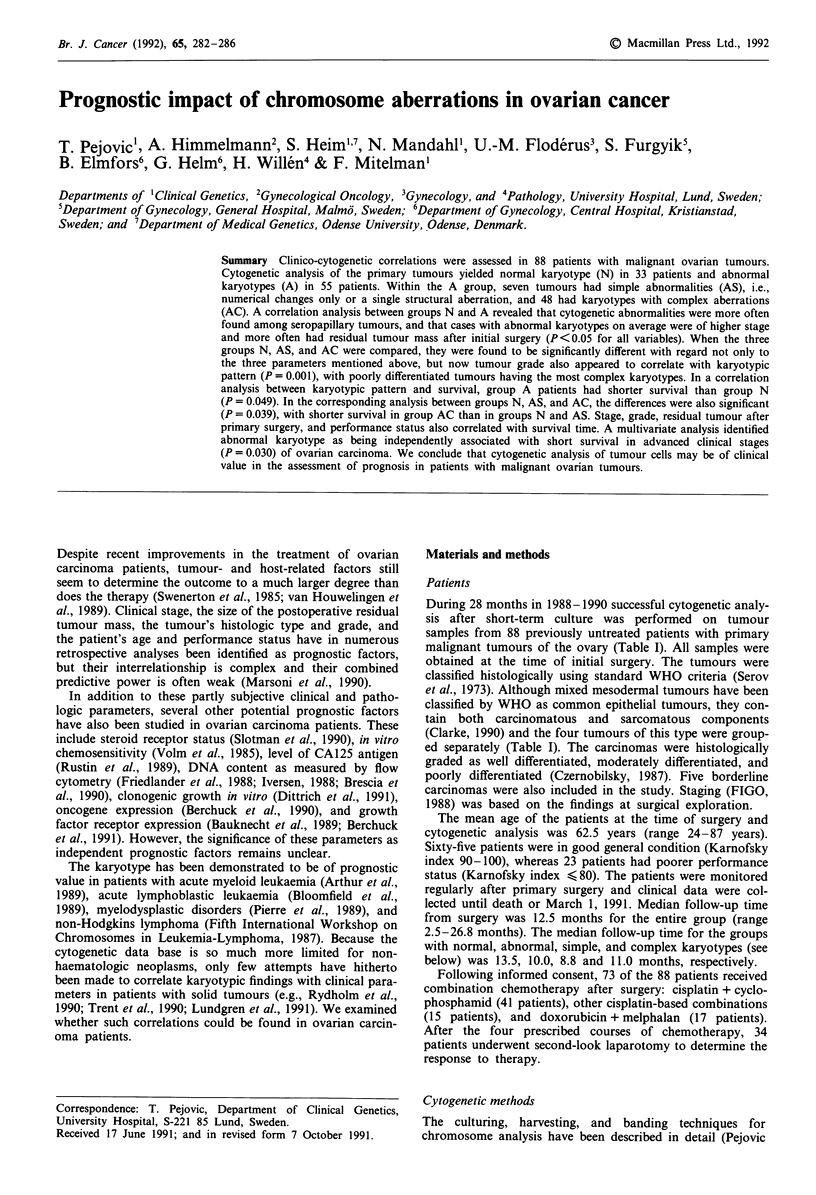

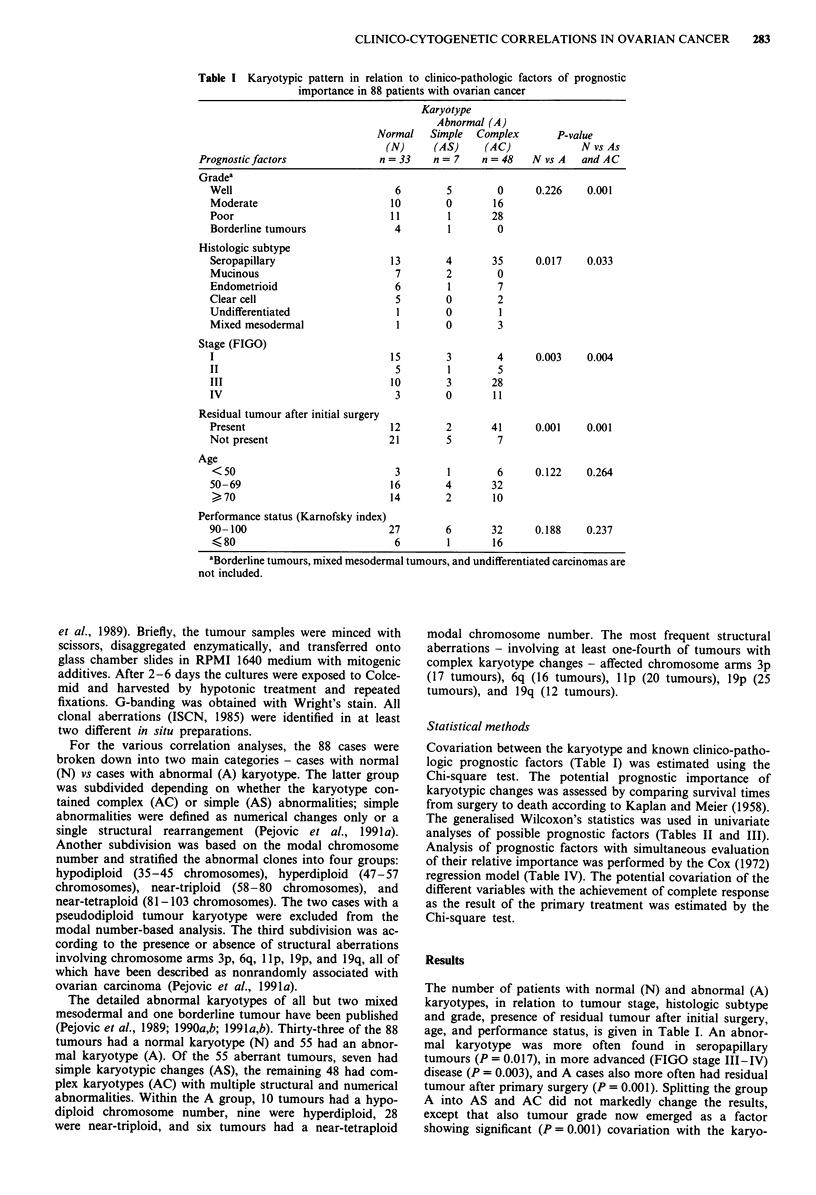

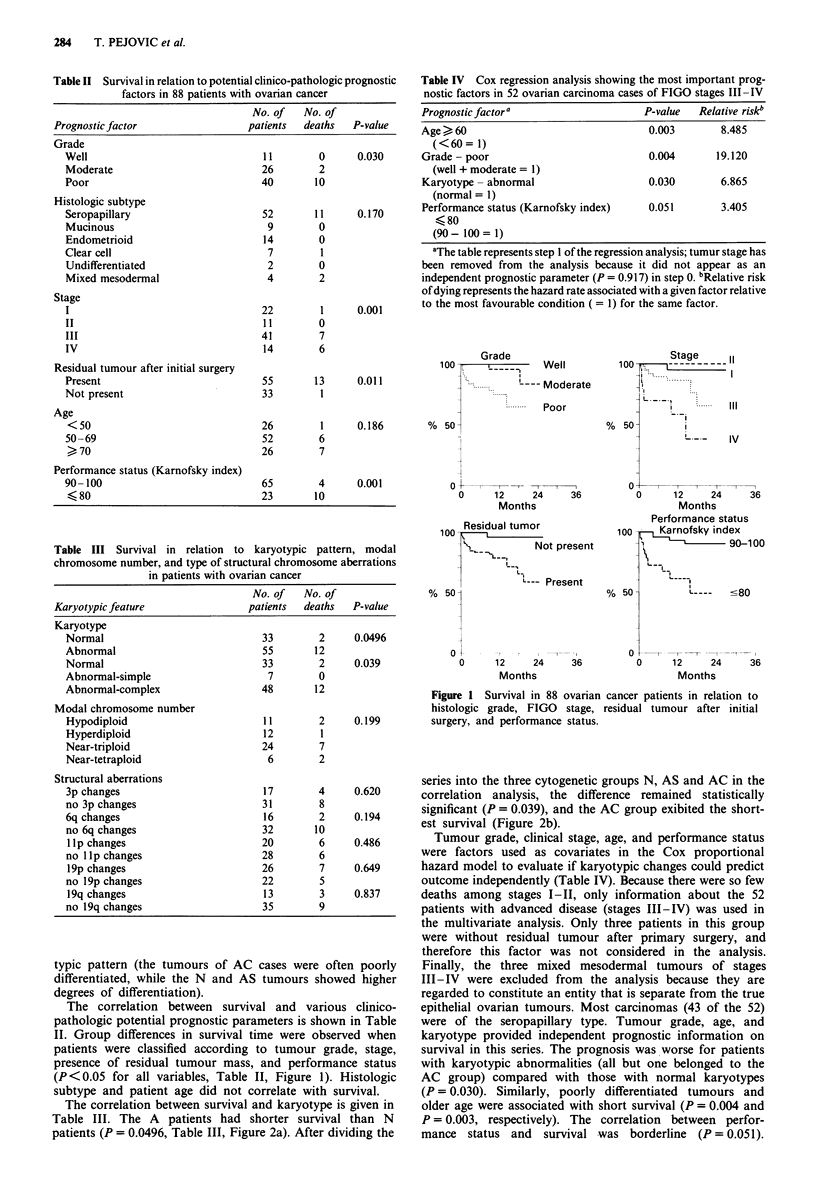

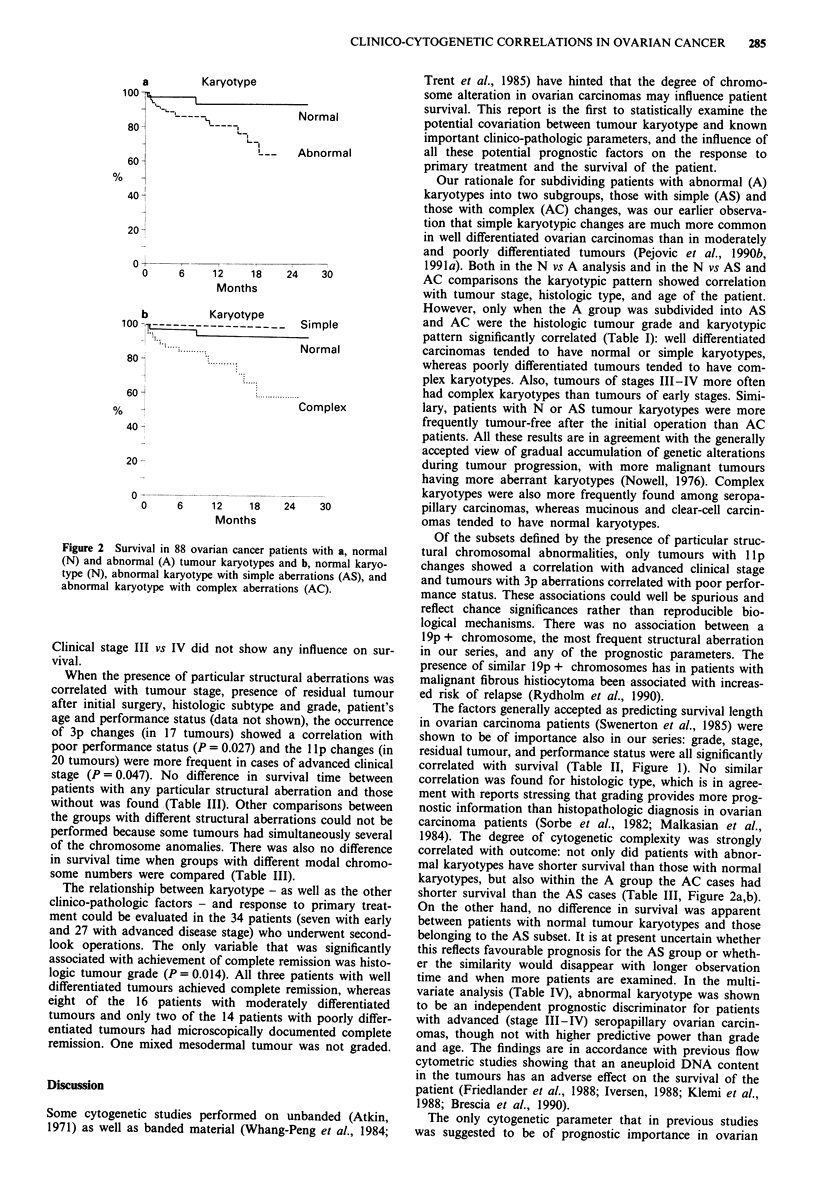

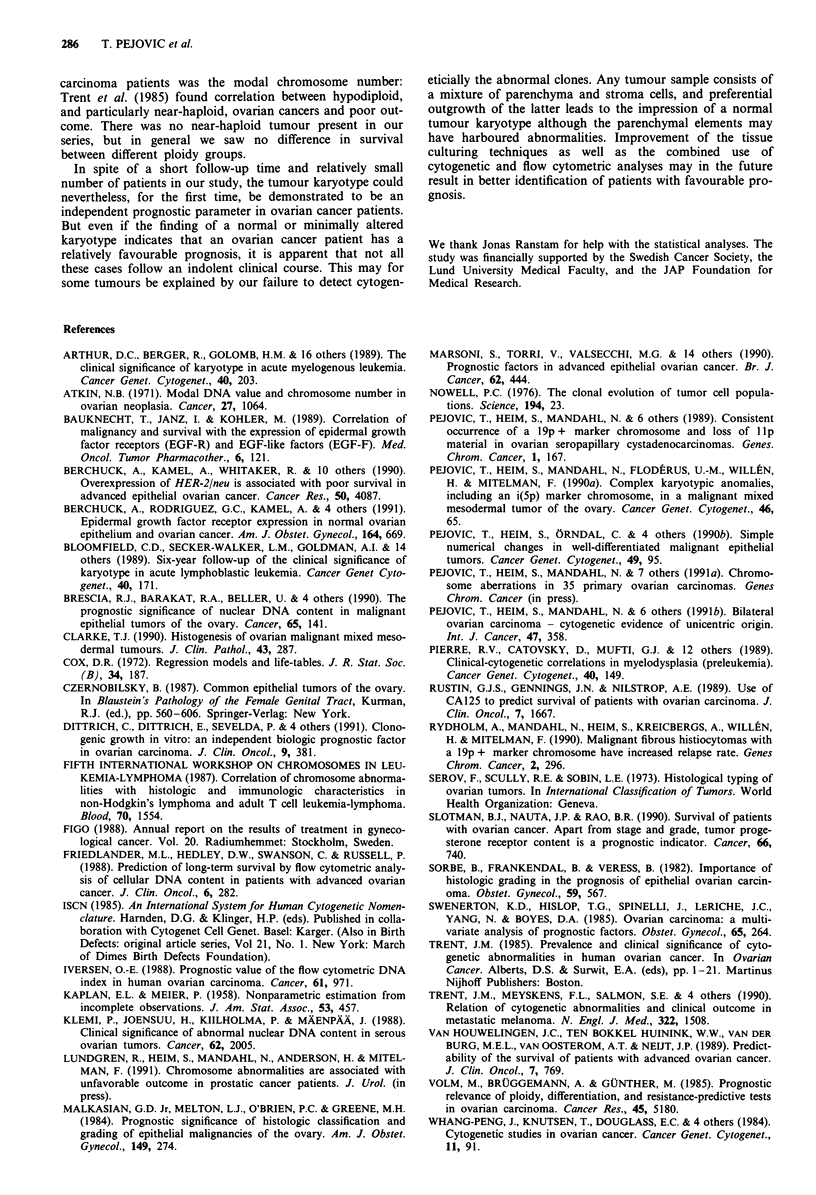


## References

[OCR_00690] Arthur D. C., Berger R., Golomb H. M., Swansbury G. J., Reeves B. R., Alimena G., Van Den Berghe H., Bloomfield C. D., de la Chapelle A., Dewald G. W. (1989). The clinical significance of karyotype in acute myelogenous leukemia.. Cancer Genet Cytogenet.

[OCR_00695] Atkin N. B. (1971). Modal DNA value and chromosome number in ovarian neoplasia. A clinical and histopathologic assessment.. Cancer.

[OCR_00699] Bauknecht T., Janz I., Kohler M., Pfleiderer A. (1989). Human ovarian carcinomas: correlation of malignancy and survival with the expression of epidermal growth factor receptors (EGF-R) and EGF-like factors (EGF-F).. Med Oncol Tumor Pharmacother.

[OCR_00707] Berchuck A., Kamel A., Whitaker R., Kerns B., Olt G., Kinney R., Soper J. T., Dodge R., Clarke-Pearson D. L., Marks P. (1990). Overexpression of HER-2/neu is associated with poor survival in advanced epithelial ovarian cancer.. Cancer Res.

[OCR_00710] Berchuck A., Rodriguez G. C., Kamel A., Dodge R. K., Soper J. T., Clarke-Pearson D. L., Bast R. C. (1991). Epidermal growth factor receptor expression in normal ovarian epithelium and ovarian cancer. I. Correlation of receptor expression with prognostic factors in patients with ovarian cancer.. Am J Obstet Gynecol.

[OCR_00714] Bloomfield C. D., Secker-Walker L. M., Goldman A. I., Van Den Berghe H., de la Chapelle A., Ruutu T., Alimena G., Garson O. M., Golomb H. M., Rowley J. D. (1989). Six-year follow-up of the clinical significance of karyotype in acute lymphoblastic leukemia.. Cancer Genet Cytogenet.

[OCR_00720] Brescia R. J., Barakat R. A., Beller U., Frederickson G., Suhrland M. J., Dubin N., Demopoulos R. I. (1990). The prognostic significance of nuclear DNA content in malignant epithelial tumors of the ovary.. Cancer.

[OCR_00725] Clarke T. J. (1990). Histogenesis of ovarian malignant mixed mesodermal tumours.. J Clin Pathol.

[OCR_00738] Dittrich C., Dittrich E., Sevelda P., Hudec M., Salzer H., Grunt T., Eliason J. (1991). Clonogenic growth in vitro: an independent biologic prognostic factor in ovarian carcinoma.. J Clin Oncol.

[OCR_00754] Friedlander M. L., Hedley D. W., Swanson C., Russell P. (1988). Prediction of long-term survival by flow cytometric analysis of cellular DNA content in patients with advanced ovarian cancer.. J Clin Oncol.

[OCR_00767] Iversen O. E. (1988). Prognostic value of the flow cytometric DNA index in human ovarian carcinoma.. Cancer.

[OCR_00775] Klemi P. J., Joensuu H., Kiilholma P., Mäenpä J. (1988). Clinical significance of abnormal nuclear DNA content in serous ovarian tumors.. Cancer.

[OCR_00786] Malkasian G. D., Melton L. J., O'Brien P. C., Greene M. H. (1984). Prognostic significance of histologic classification and grading of epithelial malignancies of the ovary.. Am J Obstet Gynecol.

[OCR_00792] Marsoni S., Torri V., Valsecchi M. G., Belloni C., Bianchi U., Bolis G., Bonazzi C., Colombo N., Epis A., Favalli G. (1990). Prognostic factors in advanced epithelial ovarian cancer. (Gruppo Interregionale Cooperativo di Oncologia Ginecologica (GICOG)).. Br J Cancer.

[OCR_00797] Nowell P. C. (1976). The clonal evolution of tumor cell populations.. Science.

[OCR_00801] Pejovic T., Heim S., Mandahl N., Elmfors B., Flodérus U. M., Furgyik S., Helm G., Willén H., Mitelman F. (1989). Consistent occurrence of a 19p+ marker chromosome and loss of 11p material in ovarian seropapillary cystadenocarcinomas.. Genes Chromosomes Cancer.

[OCR_00824] Pejovic T., Heim S., Mandahl N., Elmfors B., Furgyik S., Flodérus U. M., Helm G., Willén H., Mitelman F. (1991). Bilateral ovarian carcinoma: cytogenetic evidence of unicentric origin.. Int J Cancer.

[OCR_00807] Pejovic T., Heim S., Mandahl N., Flodérus U. M., Willén H., Mitelman F. (1990). Complex karyotypic anomalies, including an i(5p) marker chromosome, in malignant mixed mesodermal tumor of the ovary.. Cancer Genet Cytogenet.

[OCR_00829] Pierre R. V., Catovsky D., Mufti G. J., Swansbury G. J., Mecucci C., Dewald G. W., Ruutu T., Van Den Berghe H., Rowley J. D., Mitelman F. (1989). Clinical-cytogenetic correlations in myelodysplasia (preleukemia).. Cancer Genet Cytogenet.

[OCR_00834] Rustin G. J., Gennings J. N., Nelstrop A. E., Covarrubias H., Lambert H. E., Bagshawe K. D. (1989). Use of CA-125 to predict survival of patients with ovarian carcinoma. North Thames Cooperative Group.. J Clin Oncol.

[OCR_00839] Rydholm A., Mandahl N., Heim S., Kreicbergs A., Willén H., Mitelman F. (1990). Malignant fibrous histiocytomas with a 19p+ marker chromosome have increased relapse rate.. Genes Chromosomes Cancer.

[OCR_00850] Slotman B. J., Nauta J. J., Rao B. R. (1990). Survival of patients with ovarian cancer. Apart from stage and grade, tumor progesterone receptor content is a prognostic indicator.. Cancer.

[OCR_00861] Swenerton K. D., Hislop T. G., Spinelli J., LeRiche J. C., Yang N., Boyes D. A. (1985). Ovarian carcinoma: a multivariate analysis of prognostic factors.. Obstet Gynecol.

[OCR_00871] Trent J. M., Meyskens F. L., Salmon S. E., Ryschon K., Leong S. P., Davis J. R., McGee D. L. (1990). Relation of cytogenetic abnormalities and clinical outcome in metastatic melanoma.. N Engl J Med.

[OCR_00882] Volm M., Brüggemann A., Günther M., Kleine W., Pfleiderer A., Vogt-Schaden M. (1985). Prognostic relevance of ploidy, proliferation, and resistance-predictive tests in ovarian carcinoma.. Cancer Res.

[OCR_00878] van Houwelingen J. C., ten Bokkel Huinink W. W., van der Burg M. E., van Oosterom A. T., Neijt J. P. (1989). Predictability of the survival of patients with advanced ovarian cancer.. J Clin Oncol.

